# Impaired HDL Metabolism Links GlycA, A Novel Inflammatory Marker, with Incident Cardiovascular Events

**DOI:** 10.3390/jcm8122137

**Published:** 2019-12-03

**Authors:** Kayla A. Riggs, Parag H. Joshi, Amit Khera, Kavisha Singh, Oludamilola Akinmolayemi, Colby R. Ayers, Anand Rohatgi

**Affiliations:** 1 Department of Internal Medicine, The University of Texas Southwestern Medical Center, Dallas, TX 75390, USA; kayla.riggs@phhs.org (K.A.R.); Dami.Akinmolayemi@UTSouthwestern.edu (O.A.); 2 Department of Internal Medicine, Division of Cardiology, The University of Texas Southwestern Medical Center, Dallas, TX 75390, USA; parag.joshi@utsouthwestern.edu (P.H.J.); amit.khera@utsouthwestern.edu (A.K.); kavisha.singh@phhs.org (K.S.); colby.ayers@utsouthwestern.edu (C.R.A.)

**Keywords:** HDL, lipids, inflammation, atherosclerotic cardiovascular disease (ASCVD), cardiovascular events, GlycA

## Abstract

High-density lipoproteins (HDL) exert anti-atherosclerotic effects via reverse cholesterol transport, yet this salutary property is impaired in the setting of inflammation. GlycA, a novel integrated glycosylation marker of five acute phase reactants, is linked to cardiovascular (CV) events. We assessed the hypothesis that GlycA is associated with measures of impaired HDL function and that dysfunctional HDL may contribute to the association between GlycA and incident CV events. Baseline measurements of HDL cholesterol (HDL-C), HDL particle concentration (HDL-P), apoliprotein A1 (Apo A1), cholesterol efflux capacity, GlycA and high-sensitivity C-reactive protein (hs-CRP) were obtained from the Dallas Heart Study, a multi-ethnic cohort of 2643 adults (median 43 years old; 56% women, 50% black) without cardiovascular disease (CVD). GlycA was derived from nuclear magnetic resonance imaging. Participants were followed for first nonfatal MI, nonfatal stroke, coronary revascularization, or CV death over a median of 12.4 years (*n* = 197). The correlation between GlycA and hs-CRP was 0.58 (*p* < 0.0001). In multivariate models with HDL-C, GlycA was directly associated with HDL-P and Apo A1 and inversely associated with cholesterol efflux (standardized beta estimates: 0.08, 0.29, -0.06, respectively; all *p* ≤ 0.0004) GlycA was directly associated with incident CV events (adjusted hazard ratio (HR) for Q4 vs. Q1: 3.33, 95% confidence interval (CI) 1.99, 5.57). Adjustment for cholesterol efflux mildly attenuated this association (HR for Q4 vs. Q1: 3.00, 95% CI 1.75 to 5.13). In a multi-ethnic cohort, worsening inflammation, as reflected by higher GlycA levels, is associated with higher HDL-P and lower cholesterol efflux. Impaired cholesterol efflux likely explains some of the association between GlycA and incident CV events. Further studies are warranted to investigate the impact of inflammation on HDL function and CV disease.

## 1. Introduction

Although low high-density lipoprotein cholesterol (HDL-C) is considered an atherosclerotic cardiovascular disease (ASCVD) risk factor, contemporary epidemiological studies suggest that HDL particle concentration and HDL function better reflect HDL metabolism, and are better predictors of ASCVD [1,2,3,4]. In particular, cholesterol efflux capacity (CEC) as a measure of reverse cholesterol transport has been shown to inversely associate with prevalent and incident ASCVD in both low-risk and high-risk cohorts, independent of HDL-C and HDL particle concentration (HDL-P) [5]. Similarly, HDL particle concentration is also inversely associated with ASCVD, independent of HDL-C [2].

Chronic inflammation accelerates atherosclerosis, presumably in part by modifying HDL and its ability to promote reverse cholesterol transport [6,7,8,9]. GlycA is a circulating biomarker that reflects inflammation via glycosylated acute phase reactants and is directly associated with incident ASCVD [10,11,12,13,14,15]. GlycA predicts cardiovascular mortality, but the association was attenuated after adjustment for cardiovascular risk factors. Further, a recent meta-analysis also reported that GlycA is significantly associated with all-cause mortality [16].

GlycA is measured through nuclear magnetic resonance assay reflecting the mobile glycan residues from N-acetyl methyl group attachments added in the inflammatory setting. GlycA is modestly correlated with high-sensitivity C-reactive protein (hs-CRP) [12,17]. The most prominent contributions to the GlycA signal are alpha1-antitrypsin, haptoglobin, alpha1-antichymotrypsin, alpha1-acid glycoprotein, and transferrin [17]. These proteins are all associated with HDL in multiple studies, except transferrin [18,19,20]. In the setting of inflammation, protein glycan structures undergo modification, which can result in association with different receptors and changes in function [21,22]. For example, the protein composition of HDL differs in those with cardiovascular disease compared to those without cardiovascular disease [23]. hs-CRP is associated with impaired HDL cholesterol efflux [24]. Whether dysfunctional HDL is associated with this aggregated inflammatory biomarker, GlycA, is unknown. Furthermore, though inflammation and dysfunctional HDL have been linked in preclinical investigations, it remains unknown whether dysfunctional HDL explains part of the link between inflammation and ASCVD in humans. 

We assessed the hypothesis that GlycA is associated with impaired HDL function measures and that the association between GlycA and incident cardiovascular (CV) events is partially explained by dysfunctional HDL. We tested this hypothesis in the multiethnic, population-based Dallas Heart Study (DHS) by investigating cross-sectional associations between GlycA with multiple HDL parameters and longitudinal associations with incident ASCVD events.

## 2. Experimental Section

### 2.1. Study Design

The Dallas Heart Study is a multiethnic, population-based cohort of Dallas County residents aged 30 to 65 years [25]. There was intentional oversampling of black persons in the DHS, to comprise 50% of the cohort. Persons with a history of cardiovascular disease (self-reported history of myocardial infarction, stroke, heart failure, arterial revascularization, or arrhythmia), chronic kidney disease stage V or self-reported dialysis were excluded from the study, as well as individuals without values of GlycA. The final study sample included 2643 individuals. This research was approved by the UT Southwestern Medical Center Institutional Review, and all participants provided informed written consent.

### 2.2. Assessment of HDL Parameters and GlycA 

Participant blood collected in a fasting state at baseline through venipuncture was placed in ethylenediamine tetraacetic acid (EDTA) tubes and stored at four degrees Celsius for less than 4 h. Then, blood samples were centrifuged, and plasma was removed and stored at −70 degrees Celsius. 

Traditional lipids were measured through previously described methods [25]. Cholesterol efflux capacity was measured through the efflux of fluorescence-labeled cholesterol (boron dipyrromethene difluoride reagent, BODIPY) from J774 macrophages to apolipoprotein B depleted plasma [5]. Apolipoprotein A1 (Apo A1), HDL particle concentration and size, and GlycA were measured by nuclear magnetic resonance (NMR) spectroscopy (LabCorp, Inc.; formerly LipoScience, Raleigh, SC, USA). GlycA is measured through detection of glycosylation of N-acetyl methyl groups of specific serum proteins (predominately alpha1-acid glycoprotein, haptoglobin, alpha1-antitrypsin, alpha1-antichymotrypsin and transferrin) by NMR to calculate the concentration of GlycA in micromol/L [11].

### 2.3. Clinical End Points

The primary end point of the study was incident atherosclerotic cardiovascular disease (ASCVD), including first nonfatal myocardial infarction, nonfatal stroke, coronary revascularization, or CV death over a median of 12.4 years (*n* = 197). A secondary end point, total cardiovascular disease (CVD), includes ASCVD and hospitalization for congestive heart failure, atrial fibrillation, or peripheral artery disease. Death from cardiovascular causes was defined by codes I00 to I99 in the International Classification of Diseases, 10th Revision, from the National Death Index. Nonfatal cardiovascular events data were obtained from the annual detailed surveying of subjects and hospitalization admissions were tracked quarterly through the Dallas-Fort Worth Hospital Council Data Initiative database. All end points were adjudicated by two cardiologists who were blinded to all exposure variables. The vital status of participants was reviewed through 31 December 2013 [26].

### 2.4. Statistical Analysis 

GlycA was the main exposure variable and was assessed for relationships with demographics and risk factors as both a continuous variable (Spearman correlation coefficients) and as a categorical variable across increasing quartiles (Jonckheere–Terpstra Test). Multivariable linear regression models adjusted for age, sex, race/ethnicity, current smoking, hypertension, body mass index (BMI), waist circumference, diabetes, homeostatic model assessment of insulin resistance (HOMA-IR), non-HDL cholesterol, triglycerides, anti-hypertensive medications, and hs-CRP were used to assess the cross-sectional association between HDL parameters and GlycA levels. 

The hazard associated with baseline GlycA and incident ASCVD (the primary end point) and total CVD (secondary end point) was estimated using Cox proportional hazards models. Multivariable models included age, sex, race/ethnicity, smoking status, systolic blood pressure, BMI, waist circumference, diabetes, HOMA-IR, non-HDL-C, triglycerides and statins. The variance inflation factor between BMI and waist circumference is four, which does not suggest collinearity. hs-CRP and HDL parameters (HDL-C, HDL-P, ApoA1, and cholesterol efflux) were serially added to these models.

Two-sided P values of 0.05 or less were considered statistically significant. The statistical analysis was performed with SAS software version 9.4 (SAS, Raleigh, NC, USA). 

## 3. Results

### 3.1. Demographics

**Figure 1:**
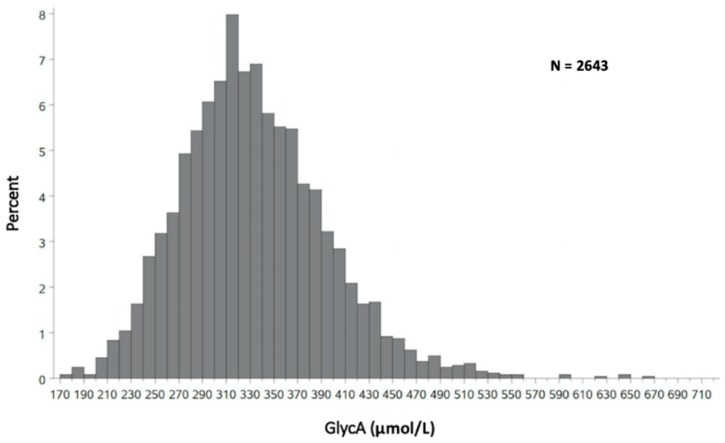
GlycA Distribution in DHS.

The mean age of study participants was 44, with 56% women and 50% blacks. GlycA was normally distributed within the DHS (Figure 1).

The median value of GlycA in DHS was 327 μmol/L (interquartile range 291 μmol/L to 369 μmol/L), with higher levels in women vs. men (*p* < 0.0001; Table 1) and significant differences by race/ethnicity (*p* < 0.0001, Table 1).

### 3.2. Univariate Correlations with GlycA

Increasing quartiles of GlycA were directly associated with age, systolic blood pressure, BMI, waist circumference, diabetes, non-HDL-C, triglycerides, and hs-CRP (Table 2). GlycA was not associated with HDL-C but was associated with increasing HDL-P. There was an inverse trend but no statistical association with cholesterol efflux (*p* = 0.06). 

Analyzed continuously in unadjusted univariate correlation analyses, GlycA was moderately correlated with hs-CRP in those (Spearman r = 0.58; *p* < 0.0001). These correlations were qualitatively higher in women vs. men (Spearman r = 0.59 for women, *p* < 0.0001; Spearman r = 0.50 for men, *p* < 0.0001) and highest in whites (Spearman r = 0.61; *p* < 0.0001).

Unadjusted associations between continuous GlycA and HDL parameters varied across parameters and by sex and ethnicity (Supplemental Table S1). GlycA correlated inversely with HDL-C (Spearman r = −0.11, *p* < 0.001) and directly with HDL-P (Spearman r = 0.14, *p* < 0.001) in women but not in men. GlycA did not correlate with efflux within any sex or ethnicity (Spearman r = −0.05–0.02, *p* = 0.07–0.74).

### 3.3. Understanding Variation in GlycA

GlycA was modeled as an outcome in adjusted linear regression models that included age, sex, race/ethnicity, current smoking, systolic blood pressure, BMI, waist circumference, diabetes, HOMA-IR, non-HDL-C, triglyceride, and antihypertensive medication. hs-CRP was added serially to this model to assess its additional contribution to variation in GlycA. Factors directly associated with GlycA included age, women, Hispanic race/ethnicity, current smoking, systolic blood pressure, BMI, waist circumference, triglycerides, and hs-CRP (all *p* < 0.05). The cardiovascular risk factor model explained 17% of the variation in GlycA, increasing to 36% of the variation when hs-CRP was included. 

HDL parameters were then individually entered into separate multivariable models, and HDL-C was included in all models. GlycA was directly associated with HDL-P and Apo A1 and inversely associated with cholesterol efflux (Table 3). Analysis of HDL subfractions revealed significant associations with medium and large HDL particles but not small HDL particles.

There was a significant interaction between HDL-P and gender with respect to GlycA (*p* for interaction < 0.001). HDL-P was associated with GlycA among women (standardized estimate 0.18, *p* < 0.0001) but not among men (*p* = ns) (Supplemental Table S2). Medium and small HDL-P were both associated with GlycA in men and women (all *p* < 0.01). 

### 3.4. GlycA, HDL Parameters, and Incident Cardiovascular Events

**Figure 2:**
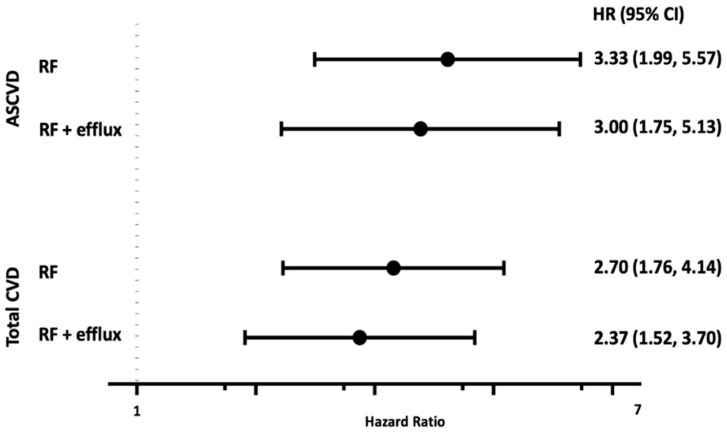
Hazard Ratios of Quartiles of GlycA (Q4 vs. Q1) derived from Cox Proportional Hazards Models adjusted for age, sex, race/ethnicity, diabetes, current smoking, systolic blood pressure, antihypertensive medications, non-high-density lipoprotein, body mass index, statin. Cholesterol efflux was serially added to the models.

Over a median of 12.4 years, there were 197 first ASCVD events. Increasing GlycA concentration was directly associated with ASCVD events in adjusted models (hazard ratio (HR) Q4 vs. Q1: 3.33, 95% confidence interval (CI) 1.99, 5.57). Further adjustment for cholesterol efflux mildly attenuated this association (HR for Q4 vs. Q1: 3.00, 95% CI 1.75 to 5.13, Figure 2). Similar attenuation was seen when analyzing GlycA and cholesterol efflux as continuous variables (Supplemental Table S3). 

Increasing quartiles of GlycA remained directly associated with ASCVD in models adjusted for HDL-P (HR for GlycA, Q4 vs. Q1: 3.46, 95% CI 2.06 to 5.84) or HDL-C (HR for GlycA, Q4 vs. Q1: 3.31, 95% CI 1.98 to 5.55). In contrast to the modest attenuation with addition of efflux, there was no attenuation with adjustment for HDL-P (HR for HDL-P, Q4 vs. Q1: 0.61, 95% CI 0.41 to 0.90). 

Over a median of 12.4 years, there were 239 total CVD events. In models adjusted for risk factors, GlycA remained directly associated with total CV events (HR for Q4 vs. Q1: 2.70, 95% CI 1.76, 4.14). Again, further adjustment for cholesterol efflux mildly attenuated this association (HR for Q4 vs. Q1: 2.37, 95% CI 1.52, 3.70); however, cholesterol efflux remained inversely associated with total CVD when adjusted for risk factors and GlycA (HR for Q4 vs. Q1: 0.69, 95% CI 0.48, 0.98). 

## 4. Discussion 

In a large, low-risk, multi-ethnic cohort, we found that GlycA as an integrated measure of inflammation associates with several HDL parameters, and impaired cholesterol efflux explains some of the relationship of GlycA with CV events. We noted gender-specific associations between GlycA and metabolic and lipid parameters and a modest correlation with hs-CRP. 

Unlike most circulating markers, which reflect single enzymes, proteins, lipids, etc., GlycA is a unique cardiometabolic marker as it directly reflects concentrations of five glycosylated acute phase reactants—alpha1-antitrypsin, haptoglobin, alpha1-antichymotrypsin, alpha1-acid glycoprotein, and transferrin [17]. Prior reports suggest that this NMR-derived marker of inflammation robustly correlates with a variety of CV phenotypes (cardiovascular disease, coronary artery disease, cardiovascular mortality, and total death), including the risk of incident CV events [10,11,12,13,14,15]. However, the relationships with cardiometabolic processes underlying these risk associations have not been fully explored.

Inflammation has been linked to disordered lipid metabolism, especially dysfunctional HDL. In particular, both acute and chronic inflammation can alter the HDL proteome and impair cholesterol transport, anti-oxidant, and other atheroprotective functions [6,7,8,9]. However, most of these observations have occurred at the preclinical level or on a limited basis among humans. Moreover, although CRP is an established generalized marker of inflammation, GlycA is distinct from CRP in that it represents multiple glycosylated proteins in response to cardiometabolic stress and perhaps is a unique reflection of the intersection between inflammation and altered protein structure and function. This is supported by the moderate but not high correlation between hs-CRP and GlycA seen in our study and previous studies. Thus, we were keenly interested in the relationship between GlycA and parameters of HDL particle composition and function.

Beyond HDL’s cholesterol load (HDL-C), total HDL particle number is a robust marker of ASCVD and outperforms HDL-C in predicting ASCVD risk [1,2,3,4]. In addition, Apo AI is the most abundant protein on HDL and associated with the majority of HDL’s atheroprotective functions. Unlike Apolipoprotein B-containing particles, which have a 1:1 ratio of Apolipoprotein B to particle number, the number of Apo AIs varies per HDL particle (2–4), resulting in only a moderate correlation between HDL particle number and Apo AI concentration [27]. Therefore, both measures of HDL particles offer complementary information on cardiometabolic risk. Lastly, the ability of HDL to promote reverse cholesterol transport is considered its most important atheroprotective function, and cholesterol efflux capacity has been the most studied aspect of this process in human epidemiologic studies, demonstrating links to prevalent and incident ASCVD [5].

The Dallas Heart Study is a population-based low risk cohort balanced by gender and enriched for African Americans, offering the opportunity to explore both gender- and race/ethnicity-specific correlates of GlycA. We discovered that several cardiometabolic risk factors including adiposity, blood pressure, and dyslipidemia are associated with higher GlycA levels. Intriguingly, several of these associations, in particular measures of adiposity, were enhanced and most prevalent among women, and either weak or non-existent among men. With respect to markers of HDL metabolism, GlycA was most strongly and directly associated with particle-related parameters (HDL-P and Apo AI) rather than cholesterol content. These relationships were most evident in non-African Americans but also varied by gender. Lastly, GlycA appeared to be inversely related to large HDL, mostly among women, mirroring the associations with adiposity.

Thus, GlycA appears to be associated with altered lipid metabolism, especially among women, and is most strongly linked to increased HDL particle number and Apo AI levels, with a trend toward smaller HDL particles. Perhaps the glycosylation of acute phase reactants reflects a milieu in which HDL particles are also modified with increased shedding of apolipoproteins and lipid-poor particles. Accounting for these relationships with HDL particle number and Apo AI levels, there emerged a modest inverse association between GlycA and cholesterol efflux, suggesting that on a per particle basis, increased glycosylation and inflammation is linked to impaired HDL function at a population level.

Having demonstrated a modest but significant link between inflammation, as reflected by GlycA and HDL metabolism, we then determined to what extent this link may explain the association between GlycA and CV events. We found a modest effect of cholesterol efflux on GlycA’s relationship with incident CV events. These findings at a population level suggest that dysfunctional HDL likely contributes to the pathways linking inflammation and CVD.

In summary, despite the independent associations of GlycA, HDL particle number, and cholesterol efflux with CV risk, markers of HDL metabolism were only modestly associated with GlycA, and cholesterol efflux was the only marker to mildly explain the link between GlycA and CV risk. Future studies may help to clarify the role of inflammation and dysfunctional HDL in CV risk by assessing higher risk cohorts with chronic CV disease and/or those undergoing acute illnesses such as acute coronary syndromes.

## Figures and Tables

**Table 1:** GlycA levels by gender and race/ethnicity in the Dallas Heart Study.

Category	Mean +/− Standard Deviation
Overall (*n* = 2643)	332 +/− 62
Men (*n* = 1152)	318 +/− 60
Women (*n* = 1491)	344 +/− 60
White (*n* = 1305)	337 +/− 64
Black (*n* = 865)	327 +/− 60
Hispanic (*n* = 417)	332 +/− 59

**Table 2:** Cardiovascular Risk Factors and high density lipoprotein (HDL) Parameters by Quartiles of GlycA (μmol/L).

	Q1 170–291 μmol/L *n* = 600 Mean (SD)	Q2 292–327 μmol/L *n* = 603 Mean (SD)	Q3 328–369 μmol/L *n* = 597 Mean (SD)	Q4 370–660 μmol/L *n* = 592 Mean (SD)	*p*-Value
Age (years)	42 (9.4)	44 (9.7)	44 (9.6)	45 (9.8)	<0.0001
Systolic Blood Pressure (mmHg)	120 (16.2)	122 (17.4)	125 (18.2)	128 (19.7)	<0.0001
BMI (kg/m^2^)	27 (5.5)	29 (5.7)	30 (7.1)	32 (8.0)	<0.0001
Waist Circumference (cm)	93 (15.0)	98 (14.8)	100 (17.0)	104 (16.6)	<0.0001
HOMA-IR	3.2 (4.1)	3.6 (3.1)	4.6 (4.8)	5.1 (4.5)	<0.0001
Non-HDL (mg/dL)	127 (39.1)	130 (39.0)	132 (39.9)	136 (41.8)	0.001
Triglycerides (mg/dL)	84 (59, 123)	91 (69, 136)	104 (72, 153)	111 (78, 162)	<0.0001
hs-CRP (mg/L)	1.9 (2.0)	3.4 (3.8)	5.2 (4.8)	9.5 (6.7)	<0.0001
HDL-C (mg/dL)	49.9 (14.6)	49.6 (14.3)	50.3 (13.9)	50.2 (15.9)	0.7600
HDL-P (μmol/L)	32.5 (5.5)	33.0 (6.1)	33.4 (5.8)	34.3 (7.5)	<0.0001
Cholesterol Efflux (normalized to pool)	1.06 (0.33)	1.01 (0.30)	1.03 (0.33)	1.02 (0.30)	0.06
Apo A1 (μmol/L)	122.1 (27.0)	125.4 (28.2)	128.2 (28.0)	132.3 (32.4)	<0.0001

BMI = body mass index, HOMA-IR = homeostatic model assessment of insulin resistance, non-HDL = non-high-density lipoprotein, hs-CRP = high-sensitivity C-reactive protein, HDL-C = high-density lipoprotein cholesterol, HDL-P = high-density lipoprotein particle concentration, Apo A1 = apolipoprotein A1. Jonckheere–Terpstra test reported for two-sided p-value. All values reported as mean (standard deviation), except for triglycerides, which are reported as median (interquartile range).

**Table 3:** Association of GlycA with HDL Parameters.

Variable	Standardized Estimate	*p*-Value
HDL-P	0.08	<0.0001
Apo A1	0.29	<0.0001
Cholesterol Efflux	−0.06	0.0004
HDL Medium	0.10	<0.0001
HDL Large	−0.07	0.02

Each row is a separate linear regression model for GlycA adjusted for age, sex, race/ethnicity, current smoking, systolic blood pressure, body mass index, waist circumference, diabetes, homeostatic model assessment of insulin resistance, high-density lipoprotein cholesterol (HDL-C), non-HDL-C, antihypertensive medication, and high-sensitivity C-reactive protein. The association displayed is for the independent addition of a single HDL parameter to this model. Only statistically significant HDL parameters are included. HDL-P = high-density lipoprotein particle concentration; Apo A1 = apolipoprotein A1.

## Supplementary Materials

The following are available online at https://www.mdpi.com/2077-0383/8/12/2137/s1, Table S1: GlycA Correlations with Variables in DHS, Table S2: Associations between HDL parameters and GlycA stratified by gender, Table S3: Cox Proportional Hazards Models of GlycA per 1 SD Association with Cardiovascular Events.
