# Supplementary material for: Impaired HDL Metabolism Links GlycA, A Novel Inflammatory Marker, with Incident Cardiovascular Events
Source: J Clin Med. 2019 Dec 3;8(12):2137. doi: 10.3390/jcm8122137 (PMC6947609; doi:10.3390/jcm8122137)
Supplement: Supplementary file 1 [file jcm-08-02137-s001.pdf]

Supplemental Material

Authors:
Kayla A. Riggs, MD
Parag H. Joshi, MD
Amit Khera, MD
Kavisha Singh, MD
Oludamilola Akinmolayemi, BS
Colby R. Ayers, MS
Anand Rohatgi, MD

The University of Texas Southwestern Medical Center Department of Internal Medicine

Corresponding Author:
Anand Rohatgi, MD, MSCS, FACC
Division of Cardiology/Department of Internal Medicine
UT Southwestern Medical Center
5323 Harry Hines Blvd.
Dallas, TX 75390-8830
Phone: 214-645-7500
Fax: 214-645-2480
Email: Anand.Rohatgi@utsouthwestern.edu

**Supplemental Table 1**. GlycA Correlations with Variables in DHS.

| | Overall | Men | Women | Black | White | Hispanic |
|---|---|---|---|---|---|---|
| hs-CRP | 0.58 | 0.50 | 0.59 | 0.57 | 0.61 | 0.50 |
| BMI | 0.30 | 0.12 | 0.39 | 0.28 | 0.33 | 0.24 |
| Waist Circumference | 0.27 | 0.13 | 0.42 | 0.30 | 0.23 | 0.17 |
| HOMA-IR | 0.27 | 0.19 | 0.34 | 0.27 | 0.28 | 0.21 |
| Triglycerides | 0.19 | 0.16 | 0.31 | 0.20 | 0.26 | 0.16 |
| Systolic blood pressure | 0.19 | 0.23 | 0.25 | 0.16 | 0.23 | 0.05 |
| Visceral fat | 0.18 | 0.16 | 0.40 | 0.23 | 0.20 | 0.04 |
| Serum glucose | 0.18 | 0.15 | 0.24 | 0.19 | 0.20 | 0.08 |
| HDL medium | 0.16 | 0.16 | 0.13 | 0.16 | 0.20 | 0.19 |
| Apo A1 | 0.14 | 0.12 | 0.06 | 0.08 | 0.17 | 0.26 |
| IL-18 | 0.13 | 0.12 | 0.18 | 0.18 | 0.11 | 0.06 |
| Age | 0.13 | 0.10 | 0.16 | 0.19 | 0.11 | 0.05 |
| MCP | 0.12 | 0.10 | 0.16 | 0.16 | 0.14 | 0.09 |
| HDL-P | 0.12 | 0.01 | 0.14 | 0.09 | 0.16 | 0.17 |
| Microalbumin creatinine ratio | 0.12 | 0.12 | 0.08 | 0.11 | 0.10 | 0.17 |
| CystatinC | 0.11 | 0.14 | 0.19 | 0.14 | 0.14 | 0.06 |
| Total Cholesterol | 0.10 | 0.08 | 0.12 | 0.06 | 0.19 | 0.06 |
| HDL small | 0.05 | -0.05 | 0.19 | 0.06 | 0.07 | 0 |
| GFR | 0.05 | 0.05 | 0.02 | -0.01 | 0.02 | 0.09 |
| LDL-C | 0.04 | 0.01 | 0.08 | 0.03 | 0.10 | -0.03 |
| HDL-C | 0 | -0.01 | -0.11 | -0.05 | 0.02 | 0.07 |
| Cholesterol Efflux | -0.03 | -0.05 | -0.01 | -0.05 | -0.02 | 0.03 |
| HDL large | -0.04 | -0.09 | -0.18 | -0.09 | -0.06 | 0.06 |
| HDL size | -0.06 | -0.04 | -0.25 | -0.10 | -0.10 | 0.07 |

hs-CRP = high-sensitivity C-reactive protein, BMI = body mass index, HOMA-IR = homeostatic model assessment of insulin resistance, HDL = high-density lipoprotein, Apo A1 = apolipoprotein A1, MCP = monocyte chemoattractant protein-1, HDL-P = high-density lipoprotein particle concentration, GFR = glomerular filtration rate, LDL-C = low-density lipoprotein concentration, HDL-C = high-density lipoprotein cholesterol.

**Supplemental Table 2**. Associations between HDL parameters and GlycA stratified by gender.

| | Variable | P-Value |
|---|---|---|
| Men | HDL-P | 0.24 |
| | Medium HDL particles | 0.003 |
| | Small HDL particles | 0.008 |
| Women | HDL-P | <0.0001 |
| | Medium HDL particles | <0.001 |
| | Small HDL particles | 0.0007 |

The interaction p-value between high-density lipoprotein particle concentration (HDL-P) and gender is <0.001.

**Supplemental Table 3.** Cox Proportional Hazards Models of GlycA per 1 SD Association with Cardiovascular Events

| | Variable | Hazard Ratio per 1 SD (95% CI) |
|---|---|---|
| ASCVD | RF | 1.38 (1.20, 1.58) |
| | RF + Cholesterol Efflux | 1.32 (1.14, 1.52) |
| | RF + HDL-P | 1.40 (1.22, 1.60) |
| Total CVD | RF | 1.27 (1.12, 1.43) |
| | RF + Cholesterol Efflux | 1.23 (1.09, 1.40) |
| | RF + HDL-P | 1.28 (1.34, 1.45) |

ASCVD = atherosclerotic cardiovascular disease, CVD = cardiovascular disease, HDL-P = high-density lipoprotein particle concentration.
